# Factors underlying the association between *Streptococcus gallolyticus*, subspecies *gallolyticus* infection and colorectal cancer: a mini review

**DOI:** 10.1017/gmb.2024.11

**Published:** 2024-11-04

**Authors:** David Michael Warner, Arunab Harish Mehta

**Affiliations:** 1 University of Cincinnati College of Medicine, Cincinnati, OH, USA; 2 Department of Hospital Medicine, University of Cincinnati Medical Center, Cincinnati, OH, USA

**Keywords:** Streptococcus gallyolyticus, colorectal carcinoma, adenoma

## Abstract

*Streptococcus gallolyticus*, subspecies *gallolyticus* (Sgg) is a gram-positive bacterium associated with infective endocarditis and colorectal cancer (CRC). Sgg has features that allow the bacterium to thrive in the colorectal tumor microenvironment and further progress the development of CRC to facilitate its survival. Sgg contains 3 pili that facilitate colonic cell adhesion and translocation through phase variation. Sgg also contains bile salt hydrolase and a bacteriocin called gallocin with substantially increased activity in bile acids, which facilitates its growth in the bile acid-rich adenomatous colorectal microenvironment. Sgg also uses tumor metabolites as an energy source. Sgg also possesses tannase, which metabolizes gallotannin to be used as a carbon source and reduces the anti-apoptotic effects of tannins, driving CRC progression. Sgg also interferes with a variety of oncogenic cell signaling pathways, including the Wnt/β-catenin pathway through mechanisms that are not fully elucidated. Increased β-catenin signaling also enhances adhesion via increased expression of the extracellular matrix and increases bile acid concentrations in the lumen through downregulation of an apical bile acid transporter. Finally, Sgg induces biotransformation of toxic substrates in CRC cells, which leads to formation of toxic intermediates and DNA adducts, promoting further progression of CRC.

## Introduction

*Streptococcus gallolyticus*, subspecies *gallolyticus* (Sgg), previously known as *Streptococcus bovis* biotype 1, is a gram-positive species of bacteria that can colonize the gastrointestinal tract as part of the gut microbiome. Sgg is associated with bacteremia, infective endocarditis and colorectal cancer (CRC). About 65% of patients with infectious endocarditis attributed to Sgg have concomitant adenomas or carcinomas detected via colonoscopy (Boleij et al., 2011a). Sgg is 5 times more likely to be isolated from fecal cultures obtained from patients with CRC compared to control human subjects (Klein et al., 1977). The association between Sgg and CRC underlies the recommendation to perform a colonoscopy on patients with positive Sgg blood cultures. The mechanism underlying the association between Sgg and CRC is not fully understood, but substantial progress has been made recently.

Tjalsma et al. (2012) proposed a passenger-driver model as a theory explaining the role of bacteria in the pathogenesis of CRC. In this model, passenger bacteria are members of the gut microbiome that gain a competitive advantage in the tumor microenvironment. Driver bacteria are members of the gut microbiome that induce epithelial DNA damage leading to the development of CRC. Sgg appears to function as both a passenger and a driver bacterium, possessing pro-carcinogenic capabilities such as directly causing DNA damage and inducing changes towards a more tumor-prone microenvironment. This mini review aims to highlight the work that has been done to enhance our understanding of the mechanisms underlying the association between Sgg and colorectal carcinoma.

## Methods

The PubMed database was utilized to search for articles on the role of Sgg in the formation of CRC. “*Streptococcus gallolyticus* colon cancer” was the primary search term used to identify articles to review. Many articles were also included if they were cited in research articles that appeared in the PubMed database. Articles were considered for review if they further elucidated the mechanisms underlying the association between Sgg and CRC, were written within the last 25 years, and a full text version was available. In total, 39 articles were evaluated and included in this mini review out of 76 search results.

## Factors promoting the adhesion, translocation, and immune evasion of Sgg

One of the first steps in the pathogenesis of Sgg-associated CRC is the preferential adhesion of Sgg to human cancer cell lines. This preferential adhesion of Sgg is aided by the displacement of Type IV collagen, which occurs in the adenomatous colorectal environment (Boleij et al., 2011b; Yantiss et al., 2001). Sgg possesses a few adaptations that allow its adhesion to human colon cancer cells.

First, Sgg possesses three pilus loci; *pil*1, *pil*2, and *pil*3 (Danne et al., 2011). The *pil*1 and *pil*3 loci in particular have been associated with the opportunistic colonization of Sgg in the adenomatous colorectal environment (Sillanpää et al., 2009). The *pil*1 locus encodes a collagen-binding adhesin, Acb, which binds in a dose-dependent manner to Types I, IV, and V collagen. Acb facilitates adhesion to Type IV collagen in the basal lamina of the colonic epithelium and plays a key role in the pathogenesis of infectious endocarditis. Additionally, Pil3 binds to mucin and fibrinogen on healthy and carcinomatous colonic epithelia through its N-terminal domain (Martins et al., 2016). Pil3 binds to mucin 5AC, which is a protein normally found in gastric tissue but can also be found in certain colonic polyps, especially larger adenomas with a moderately villous histology and dysplasia (Martins et al., 2016; Bartman et al., 1999). The binding of Pil3 to fibrinogen has been thought to contribute to the pathogenesis of infectious endocarditis by promoting biofilm formation and attaching to endothelial cells and platelets (Martins et al., 2016).

In addition to facilitating adhesion of Sgg to the cell surface of colonic epithelium, heterogeneous expression of Pil3 has been shown to also facilitate the translocation of Sgg across the intestinal barrier (Martins et al., 2020). At the population level, 90% of Sgg have low expression of Pil3 while 10% have high expression of Pil3. Martins et al. (2020) demonstrated that highly piliated Sgg interact with and open cell junctions between colonic epithelial cells to allow the passage of weakly piliated Sgg across the epithelial surface.

Furthermore, Sgg produces histone-like protein A, which binds to heparin sulfate proteoglycans at the cell surface of colorectal tumor cell surface and facilitates the adhesion of Sgg (Boleij et al., 2009).

Sgg also possesses a functional Type VII secretion system (T7SS). Type VII secretion systems have been well-described in mycobacteria and *Staphylococcus aureus* and demonstrated to have roles in adherence to host cells, immune escape, and competition with other bacterial species, Deletion of the T7SS in Sgg demonstrated that adhesion to HT29 cells, derived from a human colorectal adenocarcinoma cell line, decreased by 68% compared to wild-type bacteria (Taylor et al., 2021).

Finally, the Sgg pathogenicity-associated region (SPAR) chromosomal locus has recently been identified as a key factor in the colonization of Sgg in the gastrointestinal wall and may be essential for the function of the Type VII secretion system. SparF is believed to regulate the expression of the Type VII secretion system and a murine model of gut colonization demonstrated that deletion of the SPAR locus reduced the colonization of Sgg in the gastrointestinal tract (Taylor et al., 2023).

Sgg also has features that protect it from the innate immune system. Sgg has a polysaccharide capsule consisting of galactose, rhamnose, and uronic acid that protects against phagocytosis (Jans and Boleij, 2018). Sgg also exhibits phase variation of its pili through the addition and deletion of CGAGA repeats (Danne et al., 2014). In particular, phase variation of *pil*1 allows for variability in the amount of *pil*1 expression, favouring collagen-binding if expressed in high amounts and allowing for evasion of the humoral immune system if expressed in low amounts (Danne et al., 2014). Boleij et al. (2011b) demonstrated that Sgg also evades the immune system by delaying gene expression of IL-8 and IL-1β, which delays the recruitment of macrophages and buys time for Sgg to evade macrophages and enter the bloodstream, which is important in the pathogenesis of Sgg-associated infectious endocarditis. Sgg can also form biofilms on surfaces with abundant collagen, which plays a role in the development of infectious endocarditis.

## Ecological interactions in the adenoma and CRC microenvironment

Sgg possesses unique characteristics that favour its growth in the adenoma and CRC microenvironment and provide a competitive advantage against other bacteria.

High luminal bile acid content is a known risk factor for CRC. Bile acids also inhibit bacterial growth in several ways, including interfering with the lipid composition of bacterial membranes, chelating iron and calcium necessary for growth, directly damaging nucleic acids, and causing protein misfolding (Larabi et al., 2023). However, Sgg possesses a few properties that allows it to flourish in high bile acid environments, providing a competitive advantage in the colonic tumor microenvironment. First, the *bsh* gene found in Sgg encodes a bile salt hydrolase that provides resistance to bile salts in the gut lumen (Rusniok et al., 2010). Sgg also produces gallocin, a bacteriocin with increased activity in the presence of bile acids (Aymeric et al., 2018). Gallocin suppresses the growth of some commensal species closely related to Sgg, thereby giving Sgg an advantage in colonizing the colon (Aymeric et al, 2018). The production of gallocin is regulated via quorum sensing by the protein gallocin-stimulating peptide (Harrington et al., 2021).

In addition to flourishing in high bile acid environments, Sgg has also been shown to increase luminal bile acid concentration via the upregulation of Wnt/ β-catenin signaling, described in further detail below. Constitutive activation of the Wnt pathway has been shown to downregulate the Slc10A2 gene, which encodes an apical bile acid transporter (Aymeric et al., 2018). Downregulation of Slc10A2 results in an increase in secondary bile acid concentrations, significantly enhancing the activity of gallocin (Aymeric et al., 2018).

The Type VII secretion system possessed by Sgg also provides a competitive advantage by inhibiting the growth of commensal gut bacteria through the release of an LXG (leucine, any amino acid, glycine) toxin, which acts as a bacteriocin (Taylor et al., 2021). In particular, Sgg releases the LXG family protein TelE (toxin exported by Esx with LXG domain), which has been shown to have bacteriocidal activity when overexpressed in E. coli and is proposed to serve as a pore-forming toxin (Teh et al., 2023).

Another factor contributing to the colonization of Sgg in the colonic tumour microenvironment is the ability of Sgg to utilize tumour cell metabolites, including glucose, glucose derivatives, and alanine, as an energy source (Boleij et al., 2012).

## Factors promoting the development of CRC

In 1951, a case report was published, which described a patient with infective endocarditis caused by an enterococcal species who was also found to have underlying carcinoma of the sigmoid colon (McCoy and Mason, 1951). This case report led to a decades-long pursuit to discover the prevalence of this association, the identity of the implicated enterococcal species, and the mechanism underlying this association. A systematic review and meta-analysis performed by Boleij et al. (2011b) implicated Sgg as the implicated species and showed that 65% of patients with infective endocarditis attributed to Sgg also had underlying adenomas or carcinomas of the colon.

Sgg owes its name to its unique ability to decarboxylate gallate, a product of gallotannin. In fact, Sgg was first isolated from koala faeces likely due to the high gallotannin content of eucalyptus leaves. Tannins, found in wine, tea, coffee, fruits, and nuts, have important antimicrobial properties and have been shown to inhibit the growth of human colon cancer cells via the induction of apoptosis, the inhibition of pyruvate kinase, and inhibition of the JAK/STAT pathways (Cosan et al., 2009; Yang et al., 2018; Houssein et al., 2020; Oehmcke-Hecht et al., 2020; Youness et al., 2021).

However, Sgg is able to produce tannase, which hydrolyzes gallotannin to form gallate, which is then decarboxylated to a molecule called pyrogallol that can be used as a carbon source by Sgg. (Osawa and Sasaki, 2004; Rusniok et al., 2010; Pasquereau-Kotola et al., 2018). The breakdown of gallotannin reduces its anti-proliferative effects, thereby fostering the progression of CRC.

Sgg has also been shown to have more direct impacts on the progression of CRC through the interference of a variety of cell signaling pathways.

Ellmerich et al. (2000) showed that rats exposed to azoxymethane, a molecule that induces colonic adenomas and adenocarcinomas, and subsequently incubated with Sgg demonstrated increased proliferation of colonic crypts and elevated levels of colonic proliferation markers. Notably, rats that were not exposed to azoxymethane in this study did not develop hyperplastic colonic crypts, indicating that the pathogenesis of Sgg depends on the development of preneoplastic lesions. This study also showed that inflammatory cytokines were also increased in mice exposed to azoxymethane and incubated with Sgg, including the angiogenic factor IL-8. IL-8 is known to induce activation of MAP kinases, which stimulates cell proliferation (Biarc et al., 2004).

In addition to IL-8, Sgg has been shown to increase levels of IL-1β and COX-2, which is associated with an increased risk for the development of CRC (Abdulamir et al., 2010). IL-1β increases COX-2 expression, which promotes angiogenesis via increased expression of VEGF and inhibits apoptosis via production of PGE2 (Sheng et al., 2020). Sgg also has been shown to induce expression of NOS2, which produces nitric oxide and activates NFκB, which then also increases expression of VEGF, COX-2, and proinflammatory cytokines (Abu-Ghazaleh et al., 2021).

Kumar et al. (2017) demonstrated that colon cancer cells incubated with Sgg exhibited increased levels of β-catenin, c-Myc, and PCNA in a contact-dependent manner. The authors also showed that knockdown and inhibition of β-catenin eliminated the proliferative effects of Sgg. in vivo murine experiments performed by the authors also demonstrated that mice injected with Sgg demonstrated increased cell proliferation, β-catenin staining, and a higher tumour burden than mice models injected with control bacteria. These experiments provided a crucial link between Sgg and the adenoma-carcinoma sequence, which is a pathway implicated in 80% of sporadic colorectal carcinomas (Aymeric et al., 2018).

Dysregulation of the APC/β-catenin pathway is implicated as an initiating event in the adenoma-carcinoma sequence. In this pathway, the translocation of β-catenin to the nucleus initiates the transcription of genes promoting cell proliferation, such as cyclin D1 and c-Myc. The translocation of β-catenin to the nucleus is regulated by APC, which promotes the degradation of β-catenin. This pathway explains why loss-of-function mutations in APC, as occurs in familial adenomatous polyposis, increase the development of colon polyps and CRC. Wnt is a soluble factor that is another key component of β-catenin signaling. Wnt binds to the receptor Frizzled, which prevents the degradation of β-catenin by APC. The mechanism through which Sgg increases levels of β-catenin has not yet been fully elucidated though.

However, Kumar et al. (2022) discovered that the elevation of β-catenin induced by Sgg is dependent on Type I and Type VI collagen, which implicates the involvement of the extracellular matrix as a mediator of CRC cell proliferation. Type VI collagen serves as a bridge between Type I and Type II collagen and the extracellular matrix. In addition, the authors demonstrated that Sgg upregulates collagen, increasing the adhesion of Sgg to host cells and further inducing cell proliferation. The authors propose a few potential mechanisms through which increased collagen production can induce changes in the extracellular matrix and activate β-catenin, promoting cell proliferation. One mechanism involves the direct action of collagen on integrins that can regulate β-catenin. Another mechanism involves the indirect activation of integrins that can regulate β-catenin by fibronectin, which may become trapped by the increase in collagen in the extracellular matrix. Additionally, increased collagen production may influence the mechanical properties of the extracellular matrix, activating mechanotransducers, such as yes-associated protein (YAP) and transcriptional coactivator with PDZ-binding motif (TAZ), which are known to modulate the activity of β-catenin. Finally, the extracellular matrix can sequester other growth factors such as Wnt, FGF, EGF, and TGF-β, which can also influence cell signalling pathways involved in cell proliferation (Bonnans et al., 2014). Furthermore, β-catenin has been shown to stimulate the expression of the extracellular matrix, which may initiate a positive feedback mechanism that can enhance the interaction between Sgg and the extracellular matrix (Kreuser et al., 2020; Kumar et al., 2022).

One area still to be explored is the role of the Type VII secretion system in the activation of β-catenin. Taylor et al. (2021) demonstrated that while Sgg increased levels of β-catenin and PCNA in cell lines, this effect was abolished when the Type VII secretion system was deleted. Although the specific molecules implicated are unknown, the role of the Type VII secretion system represents a future direction of research that may help with the diagnosis and treatment of Sgg-associated CRC.

Finally, the induction of biotransformation by the aryl hydrocarbon receptor (AhR) is involved in the pathogenesis of Sgg-associated CRC. The AhR is a transcription factor that can be activated by more than 400 exogenous molecules (Dietrich and Kaina, 2010). The AhR induces Phase I and Phase II biotransformation reactions to protect cells against toxic substrates. Polyaromatic hydrocarbons, which can be found in a variety of chemicals, including charbroiled foods, cigarette smoke, and digoxin, are potent activators of the AhR, which then activates gene transcription of CYP1 and ALDH. While this can be beneficial, the Phase I and II biotransformation reactions induced by AhR activation can produce toxic intermediates that cause DNA adduct formation and promote carcinogenesis (Dietrich and Kaina, 2010).

An unknown component of the Sgg secretome has been discovered to potently activate the AhR and induce expression of CYP1 (Taddese et al., 2021). Utilizing an AhR inhibitor, Taddese et al. (2021) demonstrated that CYP1 induction was dependent on AhR and that induction of CYP1 by Sgg was dependent on AhR signalling. Induction of AhR and CYP1 by Sgg is observed in multiple colon cancer cell lines. To determine if the formation of DNA adducts could contribute to the pathogenesis of Sgg-associated CRC, the authors incubated Caco-2 cells with the Sgg secretome and then exposed the cells to 3-methylcholanthrene (3MC), a polycyclic aromatic hydrocarbon. CYP1 converts 3MC into the toxic intermediates dihydrodiol-3MC and 2-hydroxy-3MC, which have a higher affinity for DNA than 3MC and increase DNA adduct formation (Taddese et al., 2021; Eastman and Bresnick, 1979). The cells incubated with the Sgg secretome demonstrated an increase in DNA adduct formation and higher mutation rate compared to control cells, suggesting the role of the Sgg secretome in colorectal carcinogenesis.

## Conclusion

Sgg is a gram-positive, tannase-producing bacterium that is associated with infectious endocarditis and colorectal cancer. However, the mechanism underlying this association has not been fully elucidated. Tjalsma et al. (2012) proposed the passenger-driver model to evaluate the relationship between the gut microbiome and the development of colorectal cancer. Evidence for this passenger-driver model as it pertains to Sgg has been demonstrated through this mini review.

Sgg acts as a passenger through its ability to adhere and invade the colonic epithelium, compete with commensal gut bacteria through its unique T7SSb mechanism and produce gallocin, and evade the immune system through its polysaccharide capsule, phase variation regulation, ability to delay recruitment of macrophages, and biofilm formation.

Sgg acts an oncogenic driver through its ability to degrade oncoprotective tannins, dysregulate cell signalling pathways involved in the progression of CRC, and induce biotransformation, which causes the accumulation of toxic intermediates that cause DNA adduct formation.

The ability of Sgg to thrive in the colonic tumour microenvironment and promote the development of CRC provides evidence for the ability of Sgg to further drive the development of Sgg through a positive feedback mechanism. Further work is needed to understand the pharmacological interventions that may be able to inhibit the growth of Sgg and/or inhibit its ability to further drive the progression of CRC.

## References

[r1] Abdulami AS, Hafidh RR, and Baar FA (2010). Molecular detection, quantification, and isolation of *Streptococcus gallolyticus* bacteria colonizing colorectal tumors: inflammation-driven potential of carcinogenesis via IL-1, COX-2, and IL-8. Molecular Cancer 9, 249. 10.1186/1476-4598-9-249 20846456 PMC2946291

[r2] Abu-Ghazaleh N, Chua, WJ, Gopalan V (2021) Intestinal microbiota and its association with colon cancer and red/processed meat consumption. Journal of Gastroenterology Hepatology 36(1), 75–88. 10.1111/jgh.15042 32198788

[r3] Aymeric L, Donnadieu F, Mulet C, du Merle L, Nigro G, Saffarian A, Bérard M, Poyart C, Robine S, Regnault B, Trieu-Cuot P, Sansonetti PJ, and Dramsi S (2018). Colorectal cancer specific conditions promote *Streptococcus gallolyticus* gut colonization. Proceedings of the National Academy of Sciences of the United States of America 115(2), E283–E291. 10.1073/pnas.1715112115. 29279402 PMC5777054

[r4] Bartman AE, Sanderson SJ, Ewing SL, Niehans GA, Wiehr CL, Evans MK and Ho SB (1999) Aberrant expression of MUC5AC and MUC6 gastric mucin genes in colorectal polyps. International Journal of Cancer 80(2), 210–218. 10.1002/(sici)1097-0215(19990118)80:2<210::aid-ijc9>3.0.co;2-u 9935202

[r5] Biarc J, Nguyen IS, Pini A, Gossé F, Richert S, Thiersé D, Van Dorsselaer A, Leize-Wagner E, Raul F, Klein J and Schöller-Guinard M. (2004). Carcinogenic properties of proteins with pro-inflammatory activity from *Streptococcus infantarius* (formerly *S.bovis*), Carcinogenesis, 25(8), 1477–1484, 10.1093/carcin/bgh091 14742316

[r6] Boleij A, Dutilh BE, Kortman GA, Roelofs R, Laarakkers CM, Engelke UF and Tjalsma H (2012). Bacterial responses to a simulated colon tumor microenvironment. Molecular and Cellular Proteomics, 11(10), 851–862. https://10.1074/mcp.M112.019315. 22713208 10.1074/mcp.M112.019315 PMC3494137

[r7] Boleij A, Marleen MH, van Gelder J, Swinkels DW and Tjalsma H (2011a). Clinical importance of *Streptococcus gallolyticus* infection among colorectal cancer patients: systematic review and meta-analysis. Clinical Infectious Diseases, 53(9), 870–878. 10.1093/cid/cir609 21960713

[r8] Boleij A, Muytjens CMJ, Bukhari SI, Cayet N, Glaser P, Hermans PWM, Swinkels DW, Bolhuis A., and Tjalsma H (2011b). Novel clues on the specific association of *Streptococcus gallolyticus* subsp *gallolyticus* with colorectal cancer. The Journal of Infectious Diseases, 203(8), 1101–1109. 10.1093/infdis/jiq169 21451000

[r9] Boleij A, Schaeps RM, de Kleijn S, Hermans PW, Glaser P, Pancholi V, Swinkels DW, Tjalsma H (2009) Surface-exposed histone-like protein a modulates adherence of Streptococcus gallolyticus to colon adenocarcinoma cells. Infection and Immunity 77(12):5519–5527. 10.1128/IAI.00384-09. 19752027 PMC2786452

[r10] Bonnans C, Chou J, and Werb Z (2014). Remodelling the extracellular matrix in development and disease. Nature Reviewvs Molecular Cell Biology 15, 786–801. 10.1038/nrm3904 25415508 PMC4316204

[r11] Cosan D, Soyocak A, Basaran A, Degirmenci I, and Gunes HV (2009). The effects of resveratrol and tannic acid on apoptosis in colon adenocarcinoma cell line. Saudi Medical Journal, 30(2), 191–195. 19198704

[r12] Danne C, Dubrac S, Trieu-Cuot P and Dramsi S. (2014). Single cell stochastic regulation of pilus phase variation by an attenuation-like mechanism. PLoS Pathogens, 10(1), e1003860. 10.1371/journal.ppat.1003860 24453966 PMC3894217

[r13] Danne C, Entenza JM, Mallet A, Briandet R, Débarbouillé M, Nato F, Glaser P, Jouvion G, Moreillon P, Trieu-Cuot P and Dramsi S (2011). Molecular characterization of a *Streptococcus gallolyticus* genomic island encoding a pilus involved in endocarditis. The Journal of Infectious Diseases, 204(12), 1960–1970. 10.1093/infdis/jir666 22043018

[r14] Dietrich C and Kaina B (2010). The aryl hydrocarbon receptor (AhR) in the regulation of cell-cell contact and tumor growth. Carcinogenesis, 31(8), 1319–1328. 10.1093/carcin/bgq028 20106901 PMC6276890

[r15] Eastman A and Bresnick E (1979). Metabolism and DNA binding of 3-methylcholanthrene. Cancer Research, 39(11), 4316–4321. 498065

[r16] Ellmerich S, Schöller M, Duranton B, Gossé F, Galluser M, Klein J and Raul F (2000). Promotion of intestinal carcinogenesis by *Streptococcus bovis*, Carcinogenesis, 21(4), 753–756. 10.1093/carcin/21.4.753 10753212

[r17] Harrington A, Proutière A, Mull RW, du Merle L, Dramsi S, Tal-Gan Y (2021). Secretion, maturation, and activity of a quorum sensing peptide (GSP) inducing bacteriocin transcription in *Streptococcus gallolyticus*. mBio 12(1):e03189-20. 10.1128/mBio.03189-20. PMC8545107 33402540

[r18] Houssein M, Abi Saab W, Khalil M, Khalife H, and Fatfat M (2020). Cell death by gallotannin is associated with inhibition of the JAK/STAT pathway in human colon cancer cells. Current Therapeutic Research, Clinical and Experimental 92:100589. 10.1016/j.curtheres.2020.100589. 32714471 PMC7378856

[r19] Jans C and Boleij A (2018). The road to infection: host-microbe interactions defining the pathogenicity of *Streptococcus bovis/Streptococcus equinus* complex members. Frontiers in Microbiology, 10(9), 603. 10.3389/fmicb.2018.00603. PMC5902542 29692760

[r20] Klein RS, Recco RA, Catalano MT, Edberg SC, Casey JI. and Steigbigel NH (1977) Association of Streptococcus bovis with carcinoma of the colon. The New England Journal of Medicine, 297(15), 800–802. 10.1056/NEJM197710132971503 408687

[r21] Kreuser U, Buchert J, Haase A, Richter W and Diederichs S (2020). Initial WNT/β-Catenin activation enhanced mesoderm commitment, extracellular matrix expression, cell aggregation and cartilage tissue yield from induced pluripotent stem cells. Frontiers in Cell and Developmental Biology, 30(8), 581331. 10.3389/fcell.2020.581331 PMC7661475 33195222

[r22] Kumar R, Herold JL., Schady D, Davis J, Kopetz S, Martinez-Moczygemba, M, Murray BE, Han F, Li Y, Callaway E, Chapkin RS, Dashwood WM, Dashwood RH, Berry T, Mackenzie C and Xu Y (2017). Streptococcus gallolyticus subsp. gallolyticus promotes colorectal tumor development. PLoS Pathogens, 13(7), e1006440. 10.1371/journal.ppat.1006440 28704539 PMC5509344

[r23] Kumar R, Taylor JC, Jain A, Jung SY, Garza V and Xu Y. (2022) Modulation of the extracellular matrix by Streptococcus gallolyticus subsp. gallolyticus and importance in cell proliferation. PLoS Pathogens, 18(10), e1010894. 10.1371/journal.ppat.1010894 36191045 PMC9560553

[r24] Larabi AB, Masson HLP and Bäumler AJ (2023). Bile acids as modulators of gut microbiota composition and function. Gut Microbes, 15(1), 2172671. 10.1080/19490976.2023.2172671. 36740850 PMC9904317

[r25] Martins M, du Merle L, Trieu-Cuot P, and Dramsi S (2020). Heterogeneous expression of Pil3 pilus is critical for Streptococcus gallolyticus translocation across polarized colonic epithelial monolayers. Microbes and Infection Journal, 22(1):55–59. 10.1016/j.micinf.2019.12.001. 31837399

[r26] Martins M., Porrini C, du Merle L, Danne C, Robbe-Masselot C, Trieu-Cuot P and Dramsi S (2016). The Pil3 pilus of *Streptococcus gallolyticus* binds to intestinal mucins and to fibrinogen. Gut Microbes, 7(6), 526–532. 10.1080/19490976.2016.1239677 27656949 PMC5153612

[r27] McCoy WC and Mason JM (1951). Enterococcal endocarditis associated with carcinoma of the sigmoid; report of a case. Journal of the Medical Association of the State of Alabama, 21(6), 162–166. 14880846

[r28] Oehmcke-Hecht S, Mandl V, Naatz LT, Dühring L, Köhler J, Kreikemeyer B, and Maletzki C (2020). Streptococcus gallolyticus abrogates anti-carcinogenic properties of tannic acid on low-passage colorectal carcinomas. Scientific Reports, 10(1), 4714. https://10.1038/s41598-020-61458-5 32170212 10.1038/s41598-020-61458-5 PMC7070001

[r29] Osawa R and Sasaki E (2004). Novel observations of genotypic and metabolic characteristics of three subspecies of Streptococcus gallolyticus. Journal of Clinical Microbiology, 42(10), 4912–4913. 10.1128/JCM.42.10.4912-4913.2004 15472381 PMC522366

[r30] Pasquereau-Kotula E, Martins M, Aymeric L and Dramsi S (2018). Significance of *Streptococcus gallolyticus* subsp. *gallolyticus* association with colorectal cancer. Frontiers in Microbiology, 9, 614. 10.3389/fmicb.2018.00614 29666615 PMC5891635

[r31] Rusniok C, Couvé E, Da Cunha V, El Gana R, Zidane N, Bouchier C, Poyart C, Leclercq R, Trieu-Cuot, P., & Glaser, P. (2010). Genome sequence of Streptococcus gallolyticus: insights into its adaptation to the bovine rumen and its ability to cause endocarditis. Journal of Bacteriology, 192(8), 2266–2276. 10.1128/JB.01659-09 20139183 PMC2849448

[r33] Sheng J, Sun H, Yu FB, Li B, Zhang Y and Zhu YT (2020). The role of cyclooxygenase-2 in colorectal cancer. International Journal of Medical Sciences, 17(8), 1095–1101. 10.7150/ijms.44439 32410839 PMC7211146

[r34] Sillanpää J, Nallapareddy SR, Qin X, Singh KV, Muzny DM, Kovar CL, Nazareth LV, Gibbs R. A, Ferraro MJ, Steckelberg JM, Weinstock, GM. and Murray BE (2009). A collagen-binding adhesin, Acb, and ten other putative MSCRAMM and pilus family proteins of Streptococcus gallolyticus subsp. gallolyticus (Streptococcus bovis Group, biotype I). Journal of Bacteriology, 191(21), 6643–6653. 10.1128/JB.00909-09 19717590 PMC2795296

[r35] Taddese R, Roelofs R, Draper D, Wu X, Wu S, Swinkels DW, Tjalsma H, and Boleij A (2021). *Streptococcus gallolyticus* increases expression and activity of aryl hydrocarbon receptor-dependent CYP1 biotransformation capacity in colorectal epithelial cells. Frontiers in Cellular and Infection Microbiology 11, 740704. 10.3389/fcimb.2021.740704 34778104 PMC8579041

[r36] Taylor JC, Gao X, Xu J, Holder M, Petrosino J, Kumar R, Liu W, Höök M, Mackenzie C, Hillhouse A, Brashear W, Nunez MP, and Xu Y (2021). A type VII secretion system of Streptococcus gallolyticus subsp. gallolyticus contributes to gut colonization and the development of colon tumors. PLoS Pathogens, 17(1), e1009182. 10.1371/journal.ppat.1009182. 33406160 PMC7815207

[r37] Taylor JC, Kumar R, Xu J and Xu Y (2023). A pathogenicity locus of *Streptococcus gallolyticus* subspecies *gallolyticus*. Scientific Reports, 13, 6291. 10.1038/s41598-023-33178-z 37072463 PMC10113328

[r38] Teh WK, Ding Y, Gubellini F, Filloux A, Poyart C, Givskov M and Dramsi S. (2023). Characterization of TelE, a T7SS LXG effector exhibiting a conserved C-Terminal glycine zipper motif required for toxicity. Microbiology Spectrum, 11(4), e0148123. 10.1128/spectrum.01481-23. 37432124 PMC10434224

[r39] Tjalsma H, Boleij A, Marchesi JR, and Dutilh BE (2012). A bacterial driver-passenger model for colorectal cancer: beyond the usual suspects. Nature Reviws Microbiology, 10, 575–582. 10.1038/nrmicro2819. 22728587

[r40] Yang P, Ding GB, Liu W, Fu R, Sajid A, and Li Z (2018). Tannic acid directly targets pyruvate kinase isoenzyme M2 to attenuate colon cancer cell proliferation. Food and Function Journal, 9(11), 5547–5559. 10.1039/c8fo01161c 30259036

[r41] Yantiss RK, Goldman H, and Odze RD (2001). Hyperplastic polyp with epithelial misplacement (inverted hyperplastic polyp): a clinicopathologic and immunohistochemical study of 19 cases. Modern Pathology 14(9), 869–875. 10.1038/modpathol.3880403. 11557782

[r42] Youness RA, Kamel R, Elkasabgy NA, Shao P and Farag MA (2021) Recent advances in tannic acid (Gallotannin) anticancer activities and drug delivery systems for efficacy improvement; a comprehensive review. Molecule 26(5), 1486. 10.3390/molecules26051486 PMC7967207 33803294

